# New Ringer's lactate gel formulation on nasal comfort and humidification^☆^

**DOI:** 10.1016/j.bjorl.2018.07.004

**Published:** 2018-08-08

**Authors:** Maura Catafesta das Neves, Fabrizio Ricci Romano, Samuel Guerra Filho

**Affiliations:** a Universidade de São Paulo (USP), Hospital Universitário (HU), São Paulo, SP, Brazil; b Hospital Infantil Sabará, São Paulo, SP, Brazil; c Allergisa Pesquisa Dermato-Cosmética Ltda, Campinas, SP, Brazil

**Keywords:** Dryness, Nasal hydration, Nasal wash, Rhinitis, Nasal gel, Ringer lactate, Ressecamento, Hidratação nasal, Lavagem nasal, Rinite, Gel nasal, Ringer lactato

## Abstract

**Introduction:**

The use of saline irrigation for nasal washes is a well established procedure in the treatment of sinonasal inflammation and infection. In addition to saline solutions, Ringer's lactate is also an efficient option for nasal washes and humidification.

**Objective:**

To assess the comfort, humidification and tolerance regarding stinging sensation, provided by sodium chloride nasal gel at the concentrations of 4.5 mg/g and 6.0 mg/g through questionnaires answered by the patients.

**Methods:**

A total of 60 patients, 56 females, aged between 22 and 66 years old (mean age of 47) and 4 males, aged between 36 and 66 years (mean age of 49), were included in the study for a period of 17 days (±2 days) treatment. The patients were monitored by a general practitioner throughout the study period. They were instructed to apply each product in both nostrils twice a day during a 7-day period (±2 days). The patients were evaluated prior to the use of the first product at visit 0 (V0), after 7 days of treatment (±2 days) at visit 1 (V1), after 3 days of product discontinuation at visit 2 (V2) and after 7 days (±2 days) of treatment with the second product, in visit 3 (V3).

**Results:**

A significant difference (5% significance) was observed regarding comfort and stinging sensation between the two different concentrations; comfort was higher and stinging was lower with the 6.0 mg/g concentration gel. No difference in humidification was observed between the two treatments.

**Conclusion:**

Ringer's lactate at the concentration of 6.0 mg/g was superior to that at 4.5 mg/g for parameters comfort and stinging sensation. No statistical difference was observed between the two products regarding nasal humidification.

## Resumo

**Introdução:**

O uso de soluções salinas para lavagem nasal está consagrado no tratamento de quadros inflamatórios e infecciosos nasossinusais. Além das soluções salinas, o ringer lactato é uma importante opção tanto para lavagem quanto para a hidratação nasal.

**Objetivo:**

Avaliar a tolerabilidade (ardência e conforto) e umidificação do produto gel nasal cloreto de sódio 4,5 mg/g em relação ao ringer lactato 6,0 mg/g, por meio de questionários respondidos pelos pacientes.

**Método:**

Foram incluídos 60 pacientes, 56 mulheres (22-66 anos; média: 47 anos) e quatro homens (36-66 anos; média: 49 anos) foram incluídos no estudo de 17 dias (± 2 dias) de tratamento. Os pacientes foram supervisionados por um clínico geral durante todo o período do estudo. Os pacientes usaram os produtos com uma borrifada em cada narina duas vezes ao dia, durante sete dias (± 2 dias). As formulações foram avaliadas antes do uso do primeiro produto na visita 0 (V0), após sete dias (± 2 dias) de tratamento na visita 1 (V1), após três dias de interrupção do primeiro tratamento na visita 2 (V2) e após sete dias (± 2 dias) de uso do segundo produto na visita 3 (V3).

**Resultados:**

Foi observada diferença significante para o conforto das vias nasais, (significância de 5%), na comparação entre os tratamentos nos atributos de conforto e ardência. O conforto das vias nasais foi superior e a ardência inferior para o gel nasal ringer lactato 6,0 mg/g em comparação ao gel cloreto de sódio 4,5 mg/g. Não foi observada diferença significante para a umidificação entre os tratamentos.

**Conclusão:**

O gel ringer lactato 6,0 mg/g foi superior ao produto gel cloreto de sódio 4,5 mg/g nos quesitos conforto e ardência. Não foi observada diferença estatisticamente significante entre os tratamentos em relação à umidificação das vias nasais.

## Introduction

Inflammatory conditions of the nose and paranasal sinuses are the most prevalent group of diseases in the general population. Allergic and non-allergic rhinitis, as well as acute and chronic rhinosinusitis, result in a marked decrease in quality of life and generate significant losses in labor productivity, leisure and social activities in general.^1^

Nasal topical medications are extremely important in the treatment of inflammatory and infectious nasal and paranasal sinus diseases, allowing active substances to reach the targeted site in order to exert their clinical local action, minimizing the systemic effects.^1^

The majority of acute respiratory infections are of viral etiology, often presenting a self-limiting course. However, the associated symptoms (rhinorrhea, nasal obstruction, cough, pain and fever) are frequently a reason of high medication consumption.^2^

The use of saline solution for nasal hygiene has been recommended by many experts in rhinology, and its effect may be greater than just that of an adjuvant treatment.^1^

Nasal lavage with isotonic saline (0.9% sodium chloride) is recommended in respiratory infections aiming to help the mechanical removal of secretions and microorganisms in addition to increasing nasal permeability. It is an easy-to-perform procedure with no relevant adverse clinical effects, well tolerated, and beneficial to the organism.^2^

Sodium chloride nasal sprays, nasal gels and Ringer's lactate nasal gels are recommended to help the fluidification of secretions. They act by decreasing the mucus viscosity, promoting its fluidization and facilitating its elimination, and are usually indicated in any condition related to dryness of the nasal mucosa, such as low air humidity, exposure to air conditioning and pollution.

Currently, the nasal gel 4.5 mg/g, that is highly effective for nasal hydration, is already available on the market.^3^ However, its formulation is based on a glycolic component (propylene glycol) that produces a nasal stinging sensation immediately after its application.^3^ Aiming to increase nasal comfort maintaining the quality of the hydration, a new gel formulation based on Ringer's lactate solution was developed, which resulted in a concentration of 6.0 mg/g, without the glycolic component.^4^

This study aims to compare the tolerability (stinging sensation and comfort) and the efficacy of nasal hydration between sodium chloride nasal gel at 4.5 mg/g and Ringer's lactate nasal gel at 6.0 mg/g.

## Methods

A single-blind, comparative, monadic, sequential, randomized clinical study with a wash-out period of 3 days was carried out in November and December 2014.

The enrolled sample consisted of patients with recommendation of nasal gel application, who met the inclusion criteria and none of the exclusion criteria. The selected patients attended the visits for the evaluations required by the trial and signed the free informed consent form, in order to be included in the study.

The inclusion criteria comprised: participants of both genders; age >18 years; adherence to the study procedures and requirements; attendance to the clinic on the day(s) and time previously determined for the evaluations, and writing ability to provide the consent form to their participation.

Exclusion criteria included: concomitant use of any other nasal medication; pregnancy or breastfeeding; diabetes; immune deficiency diseases; use of systemic corticosteroids or immunosuppressants in a period not larger than 2 weeks before the beginning of the study; previous reaction to the tested product category; other diseases and licit or illicit medications that might directly could interfere with the study or endanger the volunteer's health and any other previously unmentioned conditions which, in the opinion of the investigator, could impair the study evaluation.

The sample size was determined based on the ANVISA guide to cosmetic product studies,^5^ which requires a minimum number of 30 responses for safety studies. Therefore, a sample of 60 patients was established. Patient recruitment was carried out by Allergisa, a company with a computerized and updated registry system of individuals interested in researches participation.

The sponsor company Libbs Farmacêutica LTDA provided the nasal gel samples to the researcher,^3^, ^4^ which were evaluated in standardized packages identified with a label indicating only the type of product, product code, validity and sample lot as shown in Table 1. The name of the product was not identified on the sample labels of the study. Code 43329-1 corresponded to Ringer's lactate nasal gel, at 6.0 mg/g,^4^ whereas code 43329-02 corresponded to sodium chloride nasal gel, at 4.5 mg/g.^3^

**Table 1:** Standardization of sample labels delivered to patients.

Type of product	Product code	Product lot	Product validity
Nasal gel	43329-01	LP247/14A	09/2016
Nasal gel	43329-02	14/0042	09/2016

### Study design

Patients were assessed by a general practitioner for inclusion and exclusion criteria and for their clinical status (history of diseases, allergies, blood pressure and general physical examination) at visit 0 (V0). Approved female patients underwent also to a urinary pregnancy test on V0.

After giving their informed consent and being included, the patients received one of the products to be applied to the nose for 7 ± 2 days (one spray in each nostril every 12 h). Product prescription was randomized, so that the patients did not know which product they were using.

After 7 ± 2 days the patients were evaluated at visit 1 (V1) for monitoring the onset of any adverse events, answering a questionnaire to evaluate the product.

After a 3-day interval without using any product (wash out), the patients returned for visit 2 (V2) to receive the second product to be used for 7 ± 2 days. After 7 ± 2 days the patients returned on visit 3 (V3) for the final assessment (clinical examination and new pregnancy test, for female patients). Table 2 outlines the visits and follow-up.

**Table 2:** Schedule of procedure.

Stage	V0	V1	V2	V3
Signature of term of informed consent	X			
Clinical evaluation	X	X	X	X
Pregnancy test	X			X
Evaluation of efficacy and tolerability		X		X
Product delivery	X		X	
Product return		X		X

For the analysis of the acceptability results, the study considered patients with a minimum adherence of 80% regarding the frequency of use. As the prescription consisted of twice applications a day for 7 days, 14 applications were expected as the final result. Considering the minimum adherence of 80% regarding the product application frequency, each participant should have applied at least 11 times to have their evaluations valid and accounted for the final study results.

**Figure 1:**
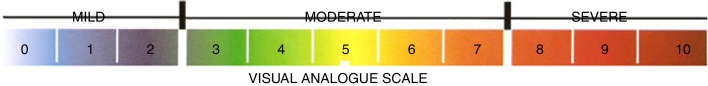
Visual analog scale – VAS (comfort and stinging).

**Figure 2:**
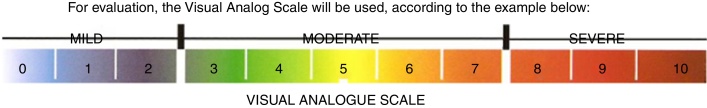
Visual analog scale – VAS (humidification).

The efficacy (humidification) and tolerability (stinging sensation and comfort) evaluation was performed through questionnaires answered by the patients. A visual analog scale (VAS) was used for each item as shown in Figure 1, Figure 2.

The investigator showed the VAS scale to each patient and asked them to indicate the point that best represented each assessed item. The investigator verified the point indicated by the patient in the figure, which ranged from 0 (zero) to 10 (ten) centimeters and noted the corresponding value. In this scale, the value of 0 (zero) represented the lowest efficacy perceived at the treatment and the value of 10 (ten) represented the greatest efficacy.

All participants who did not meet the inclusion/exclusion criteria prior to the beginning of the study or that did not agree participating before or after the informed consent form signature were considered as “selection failure”. These participants were replaced, and their data were not considered in the final report. There was no replacement for those who withdrew after receiving the product under investigation.

This study was carried out according to the principles of the Helsinki Declaration of October 2008, and the applicable regulatory requests including CNS Resolution No. 466/2012,^6^ and the principles of Good Clinical Practices (Document of the Americas and ICH E6: Good Clinical Practice).^7^ The study was approved by the institutional research ethics committee (CAAE: 37042514.8.0000.5599).

## Results

A total of 60 patients who met all the inclusion and exclusion criteria were enrolled, 56 were females, aged between 22 and 66 years (mean: 47 years) and four were males, aged between 36 and 66 years (mean: 49 years). In a complementary study, a primary skin irritation potential of products (patch test) was evaluated. In this study, none of the participants showed clinical signs of skin irritation related to the product and the latter did not induce primary skin irritation process in the study group.

The results obtained for each assessed characteristic are shown in Table 3.

**Table 3:** Mean, standard deviation and result of the comparison between treatments.

Characteristic	Ringer's lactate nasal gel 6.0 mg/g		Sodium chloride nasal gel 4.5 mg/g		*p* value
	Mean	SD	Mean	SD	
Comfort	8.6	1.7	7.7	2.1	0.003^b^
Stinging	0.2	1.0	1.5	2.7	<0.001^c^
Humidification	8.9	1.3	8.6	1.4	0.136

^a^ Significant at the 5% level (Wilcoxon test).

b Significant at the 1% level.

c Significant at the 0.1% level.

A significant difference was observed, with a significance level of 5%, when comparing the treatments regarding the characteristics of comfort and stinging sensation. The comfort of nasal application was superior with Ringer's lactate nasal gel, at 6.0 mg/g^4^ compared to sodium chloride nasal gel, at 4.5 mg/g.^3^ The stinging sensation was lower for the treatment with Ringer's lactate nasal gel, at 6.0 mg/g,^4^ compared with the nasal gel, at 4.5 mg/g.^3^ No significant difference was observed regarding nasal humidification when comparing both treatments.

**Figure 3:**
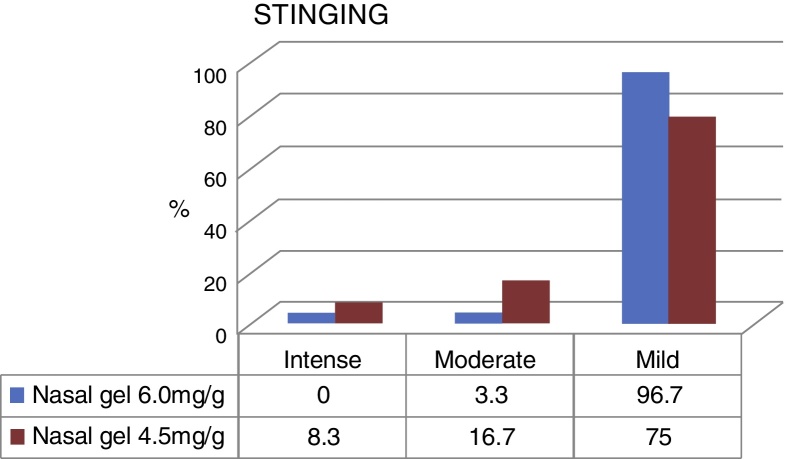
Stinging with nasal gel 6.0 mg/g^4^ × nasal gel 4.5 mg/g.^3^

Fig. 3 compares the data obtained regarding the stinging sensation for each tested product concentrations, on VAS scale. The stinging sensation was reported as moderate to severe by 15 patients who tested the sodium chloride nasal gel, 4.5 mg/g^3^ and moderate for only 2 patients who tested Ringer's lactate nasal gel, at 6.0 mg/g.^4^ There were no reports of intense stinging sensation among patients that used Ringer's lactate gel at the concentration of 6.0 mg/g.^4^

**Figure 4:**
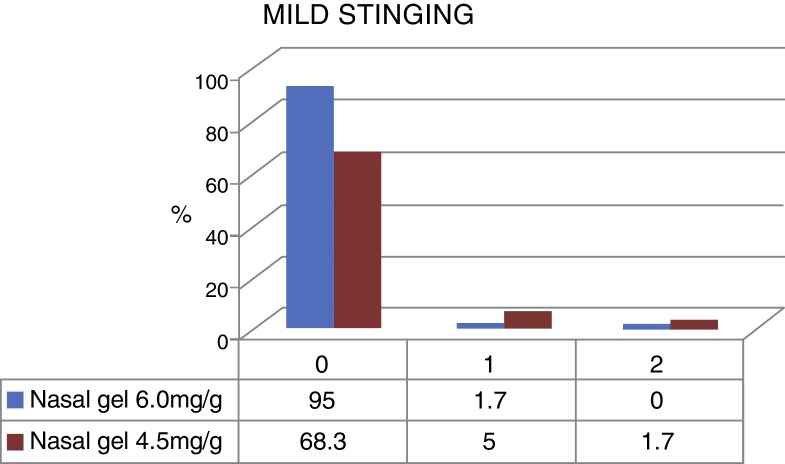
Division of mild stinging reports for Ringer's lactate nasal gel 6.0 mg/g^4^ × sodium chloride nasal gel 4.5 mg/g.^3^

The stinging sensation was reported as mild by 58 patients who tested for the concentration of 6.0 mg/g^4^ and by 45 patients that tested for the concentration of 4.5 mg/g^3^ (Fig. 3). The stinging sensation was reported as ZERO (included as mild in the VAS scale) by 57 patients (95%) tested for ringer's lactate gel at a concentration of 6.0 mg/g^4^ vs. 41 patients (68.3%) who tested the sodium chloride nasal gel at a concentration of 4.5 mg/g^3^ (Fig. 4).

**Figure 5:**
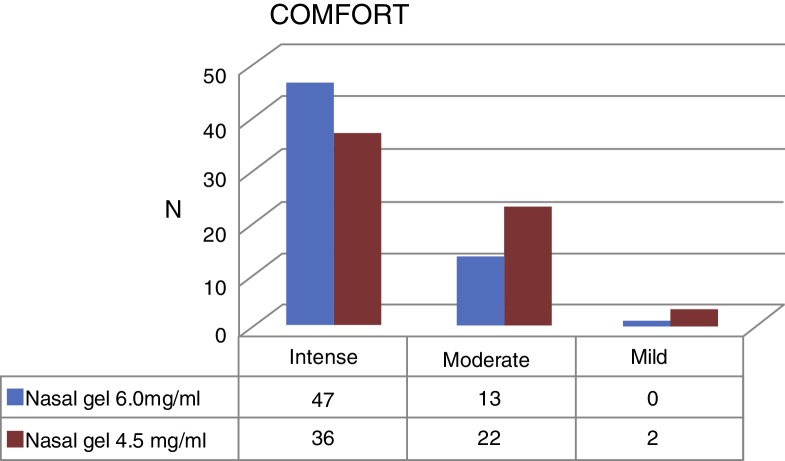
Comfort with nasal gel 6.0 mg/g^4^ × nasal gel 4.5 mg/g.^3^

Regarding the comfort, Ringer's lactate gel at 6.0 mg/g^4^ provided intense comfort for 47 patients (78.3%) and moderate for 13 patients, (21.7%) while the concentration of 4.5 mg/g^3^ provided intense comfort for 36 (60%) patients, moderate for 22 (36.7%) and mild for 2 patients (3.3%) (Fig. 5).

**Figure 6:**
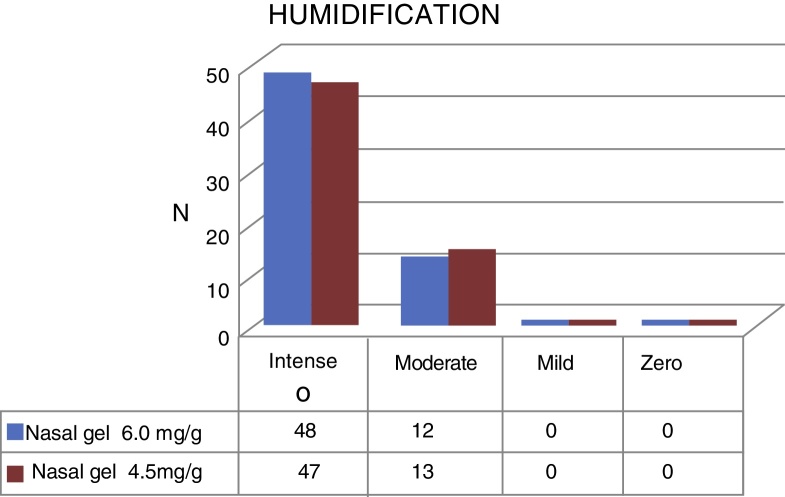
Humidification with Ringer's lactate nasal Gel 6.0 mg/g^4^ × sodium chloride nasal gel 4.5 mg/g.^3^

Regarding humidification, the concentration of 6.0 mg/g^4^ of Ringer's lactate gel provided intense humidification for 48 (80%) patients and moderate for 13 (20%), while the concentration of 4.5 mg/g^3^ of the sodium chloride nasal gel provided intense humidification for 47 (78.4%) and moderate for 13 patients (21.6%), as shown in Fig. 6.

**Figure 7:**
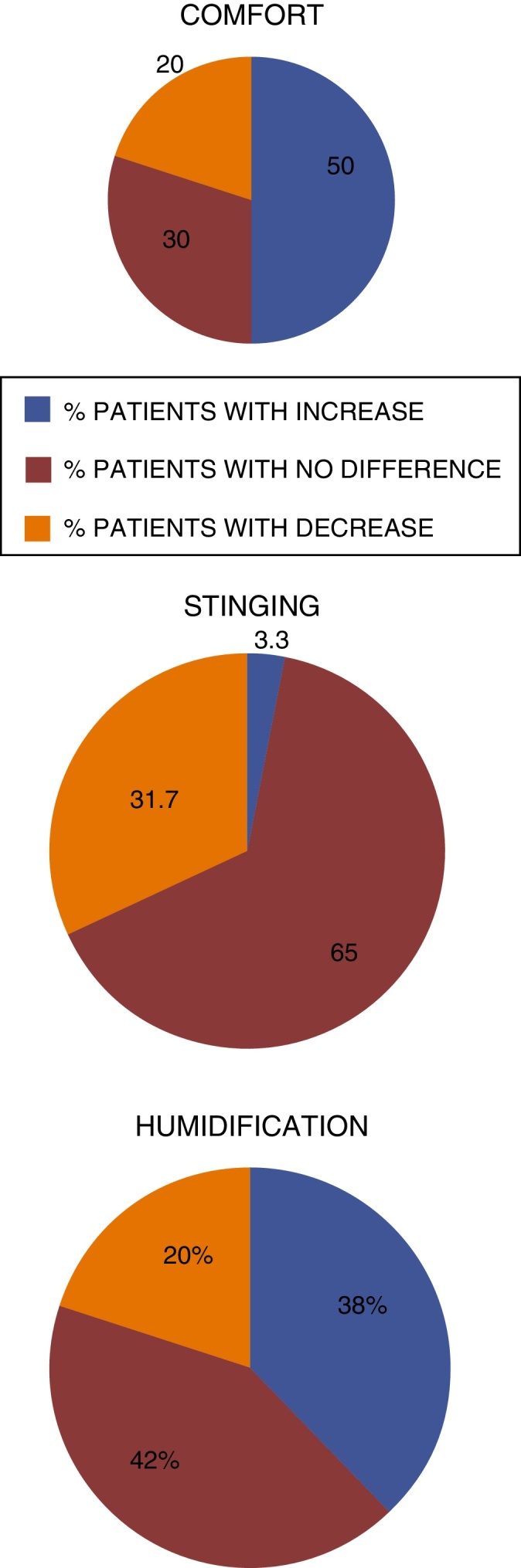
Percentage of participants with increase, no difference and with reduction of characteristics during the treatment with Ringer's lactate nasal gel 6.0 mg/g^4^ × nasal gel 4.5 mg/g.^3^

Fig. 7 compares the results obtained for each characteristic evaluated for each nasal gel concentration.

## Discussion

The use of isotonic saline solutions for nasal cleaning is well disseminated and clinically established for more than 70 years as a practical, effective and well tolerated method of promoting well-being and nasal physiology maintenance.^8^ These solutions are recommended as complementary therapy in cases of acute and chronic rhinosinusitis, rhinitis, post-nasal drip, septum perforation and postoperative care.^9^, ^10^

Their action mechanism involves the mechanical removal of mucus, crusts and nasopharyngeal secretion and alteration of the viscoelastic characteristics of the mucus (hydrating it through osmotic gradient and altering the bonds between glycoproteins), which facilitates the mucociliary clearance.^11^

There is no clear evidence in the literature of which type of saline solution is the most appropriate for this function. However, the study of Boek^8^ showed that nasal Ringer's lactate isotonic solution is more appropriate than other nasal saline solutions.

According to the literature, every 100 mL of ringer's lactate solution contains 600 mg of sodium chloride, 310 mg of sodium lactate, 30 mg of potassium chloride and 20 mg of calcium chloride dihydrate.^12^, ^13^, ^14^ This formulation provides an increase in the alkaline content, a fact that occurs after its metabolization into bicarbonate. Moreover, ringer's lactate solution has potassium and calcium at concentrations that are similar to the ionized concentrations found in normal blood plasma. To maintain electrical neutrality, the solution has lower sodium level than that found in isotonic saline solution or plasma. The combination of all salts results in an osmolarity similar the blood, which makes this solution isotonic, with a mean pH of 6.75 (6.0–7.5).^12^, ^13^, ^14^

Ringer's lactate nasal gel has a concentration of 6 mg/g due to the predominant presence of sodium chloride in its composition in solid form (expressed in mg/g) and becomes isotonic with the presence of other ions in its formulation.

This evidence was confirmed by other investigators^15^, ^16^, ^17^, ^18^ when comparing a ringer's lactate solution with a 0.9% saline solution, demonstrating a statistically significant improvement in mucociliary clearance with the ringer's lactate solution compared to other saline solutions.

Ringer's lactate solution^12^, ^13^, ^14^ shows evidence of being an excellent nasal humectant, possibly because it causes less irritating effects on the respiratory mucosa, even when compared to the “physiological” 0.9% NaCl saline solution in experimental^19^ and clinical studies.^17^, ^20^, ^21^ As a limitation of the present study, we should mention the enrollment of female participants in the study sample since the menstrual cycle period was not evaluated.

Azzam^16^ emphasizes that although the isotonic saline solution is recommended for repeated use several times a day and that is very important for the mechanical removal of crusts and secretions and nasal lavage, its hydrating power is very limited and transitory. The current concept of nasal humidification, according to Azzam,^16^ should be directed to the pharmaceutical form of “gel”, which allows a longer period of product adhesion to the nasal mucosa,^22^ and have been considered safe and effective in several clinical studies.^18^, ^23^, ^24^

The tested products comprise a widely marketed formulation (sodium chloride nasal spray, at a concentration of 4.5 mg/g^3^ and a new formulation (Ringer's lactate nasal gel, at a concentration of 6.0 mg/g).^4^

Two important modifications in the formulation differentiate the nasal gel at 4.5 mg/g^3^ from the Ringer's lactate nasal gel at 6.0 mg/g.^4^ First, the presence of more ions in the Ringer's lactate solution is the base of the nasal gel at a concentration of 6.0 mg/g,^4^ which confers unique properties to this formula. Furthermore, propylene glycol, which provides the gel consistency for the nasal gel formula at 4.5 mg/g^3^ was removed from the nasal gel at 6.0 mg/g, and replaced by hydroxyethyl cellulose (HEC).^4^ HEC^25^, ^26^ is a semi-synthetic viscoelastic polymer derived from the water-soluble cellulose polysaccharide with a osmotic potential, currently used in ophthalmologic products,^26^ in oral capsules and tablets,^25^ to stabilize liposoluble drug solutions, as a plasma expander, as a hemostatic agent and as surface hydrating agent.^23^, ^25^

In clinical studies, the use of HEC as a vehicle for nasal solutions has been reported as very safe. Several published studies have shown that nasal solutions with this component do not have significant side effects.^15^ This suitability has promoted greater comfort, minimizing the stinging sensation that is frequently experienced with the previous formulation.

The results showed greater tolerability when using Ringer's lactate gel at 6.0 mg/g^4^ when compared to the currently marketed nasal gel at 4.5 mg/g^3^ providing greater comfort and lower incidence of stinging sensation, which were statistically significant. Approximately 95% of the patients reported no stinging sensation when applying the nasal gel at a concentration of 6.0 mg/g^4^ when compared to 68% of the patients using the nasal gel at a concentration of 4.5 mg/g.^3^ Moreover, the patients showed the same perception of nasal hydration with both products used in this study, which indicates similar efficacy between the formulations.

The currently marketed formulation has multiple clinical uses, including postoperative nasal surgery,^15^ in addition to several clinical conditions such as rhinitis, sinusitis, use of continuous positive air pressure (CPAP), among other mucosal dryness conditions.^3^, ^16^, ^24^ A new formulation without the glycolic component (responsible for some stinging) and based on ringer's lactate solution will provide greater comfort and hydration many clinical conditions.^4^, ^16^, ^17^, ^20^

## Conclusion

Ringer's lactate gel is an alternative for nasal humidification by conferring nasal comfort with high level of tolerance and minimal stinging sensation. Ringer's lactate gel formulation at 6.0 mg/g^4^ was significantly superior to that at 4.5 mg/g sodium chloride nasal gel formulation^3^ regarding comfort and stinging, while the humidification sensation was high for both concentrations.

## Conflicts of interest

The authors declare no conflicts of interest.
